# Crystal structure of bis­(aceto­phenone 4-benzoyl­thio­semicarbazonato-κ^2^ *N* ^1^,*S*)nickel(II)

**DOI:** 10.1107/S2056989016006873

**Published:** 2016-04-29

**Authors:** Faraidoon Karim Kadir, Mustaffa Shamsuddin, Mohd Mustaqim Rosli

**Affiliations:** a Department of Chemistry, Faculty of Science, Universiti Teknologi Malaysia, 81310 UTM Johor Bahru, Johor, Malaysia; b Department of Chemistry, School of Science, Faculty of Science & Education, University of Sulaimani, Kurdistan Region, Iraq; c Centre for Sustainable Nanomaterials, Universiti Teknologi Malaysia, 81310 UTM Johor Bahru, Johor, Malaysia; d X-ray Crystallography Unit, School of Physics, Universiti Sains Malaysia, 11800, USM, Penang, Malaysia

**Keywords:** crystal structure, thio­semicarbazone, nickel(II), anagostic inter­actions, C—H⋯O inter­actions

## Abstract

In the asymmetric unit of the title complex, [Ni(C_16_H_14_N_3_OS)_2_], the nickel ion is tetra­coordinated in a distorted square-planar geometry by two independent mol­ecules of the ligand which act as mononegative bidentate *N*,*S*-donors and form two five-membered chelate rings. The ligands are in *trans* (*E*) conformations with respect to the C=N bonds. The close approach of hydrogen atoms to the Ni^2+^ atom suggests anagostic inter­actions (Ni⋯H—C) are present. The crystal structure is built up by a network of two C—H⋯O inter­actions. One of the inter­actions forms inversion dimers and the other links the mol­ecules into infinite chains parallel to [100]. In addition, a weak C—H⋯π inter­action is also present.

## Abstract 2

In [Ni(C_16_H_14_N_3_OS)_2_], the nickel ion is tetra­coordinated in a square-planar geometry by two independent mol­ecules of the ligand which act as mononegative bidentate *N*,*S*-donors and form two five-membered chelate rings. Close approach of hydrogen atoms to Ni^2+^ suggests anagostic inter­actions (Ni⋯H—C) are present.

## Chemical context   

Thio­semicarbazones containing N and S donor atoms have been widely used in metal coordination chemistry due to their structural flexibility and versatility (Pelosi *et al.*, 2010 ▸; Yousef *et al.*, 2013 ▸; Jagadeesh *et al.*, 2015 ▸). The chemistry of transition metal complexes of thio­semicarbazones has gained significant attention due to their potential medicinal applications (Pelosi *et al.*, 2010 ▸; Li *et al.*, 2012 ▸; Manikandan *et al.*, 2014 ▸). The variable mode of binding of thio­semicarbazone towards nickel has encouraged us to explore its coordination chemistry further since nickel has the ability to take up different coord­ination environments. Nickel complexes are known to catalyse carbon–carbon cross-coupling and other reactions (Suganthy *et al.*, 2013 ▸; Wang *et al.*, 2014 ▸).
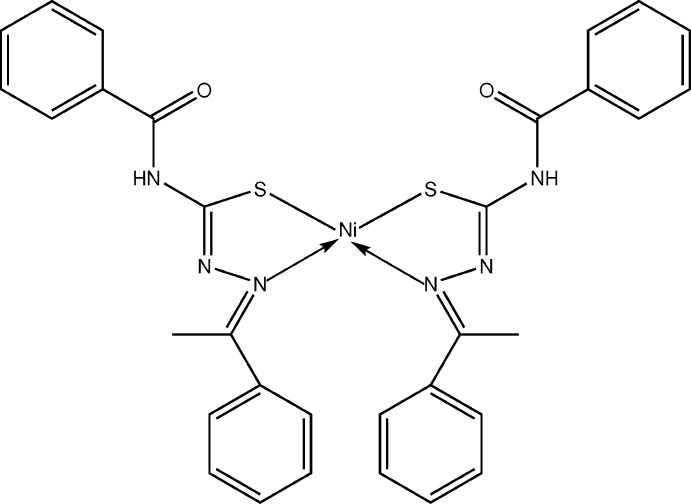


## Structural commentary   

**Figure 1:**
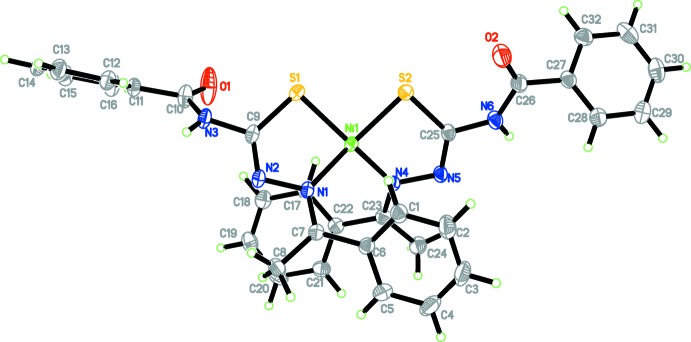
The mol­ecular structure of (I) showing 50% probability displacement ellipsoids. H atoms are shown as spheres of arbitrary radius.

The mol­ecular structure of the title complex (I) with the numbering scheme is shown in Fig. 1 ▸. The nickel ion is tetra-coordinated in a square-planar geometry by two crystallographically independent mol­ecules of the ligand which act as mononegative bidentate *N*,*S*-donors and form two five-membered chelate rings. The ligands are in *trans* (*E*) conformations with respect to the C7=N1 and C23=N4 bonds, as evidenced by the torsion angles N2—N1—C7—C6 = −171.0 (2) and N5—N4—C23—C22 = −171.8 (2)°, respectively. This is in close agreement with previously reported data (Sampath *et al.*, 2013 ▸, Suganthy *et al.*, 2013 ▸). A remarkable tetra­hedrally distorted square-planar coordination geometry is shown by the nickel metal ion, with the two ligands displaying a less common *cis N*,*S*-chelation mode (de Oliveira *et al.*, 2014 ▸). The Ni—S and Ni—N bond lengths (Table 1 ▸) and the N1—Ni1—S2 and N4—Ni1-S1 bond angle of 159.86 (7) and 159.67 (7)°, respectively, confirm the distortion from a typical coordination geometry.

Upon chelation to the Ni^II^ ion, the ligands underwent deprotonation from the tautomeric thiol­ates and their negative charges are delocalized over atoms N1–N2–C9–S1 and N4–N5–C22–S2. Consequently, the bond lengths S1—C9 in one ligand and S2—C25 in the other ligand are 1.728 (3) and 1.735 (3) Å, respectively, which are consistent with single-bond character (Sankaraperumal *et al.*, 2013 ▸). Furthermore, the Ni—N [1.922 (2) and 1.928 (2) Å] and Ni—S bond lengths [range 2.1489 (10) and 2.1518 (10) Å] are consistent with those in similar reported compounds. The S—C [1.728 and 1.735 (3)Å] and N—C [1.293 (3) and 1.294 (3) Å] bond lengths of the ligand are consistent with literature values (Sankaraperumal *et al.*, 2013 ▸, de Oliveira *et al.*, 2014 ▸).

**Figure 2:**
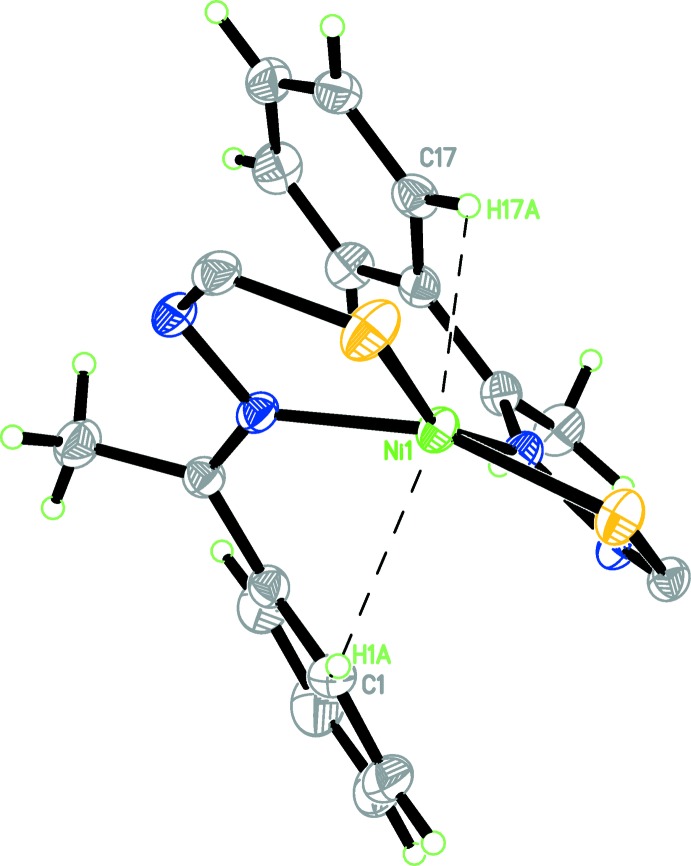
Two anagostic inter­actions (dashed lines) between the nickel(II) ion and the aromatic C—H groups.

Notably, two anagostic inter­actions in the *trans*-arrangement are observed in the title complex between the nickel(II) ion and the aromatic C—H groups (Fig. 2 ▸). The Ni1⋯H1*A* and Ni1⋯H17*A* distances are 2.616 and 2.527 Å, respectively, which are shorter than the van der Waals radii sum for Ni (1.63 Å; Bondi, 1964 ▸) and H (1.10 Å; Rowland & Taylor, 1996 ▸). In addition, the Ni1—H1*A*—C1 and Ni1—H17*A*—C17 bond angles are 109.6 and 112.7°, respectively. These observed values of contact distances and bond angles fall in the range for anagostic inter­actions reported by Brookhart *et al.* (2007 ▸). Similar observations have been reported recently by de Oliveira *et al.* (2014 ▸).

## Supra­molecular features   

**Figure 3:**
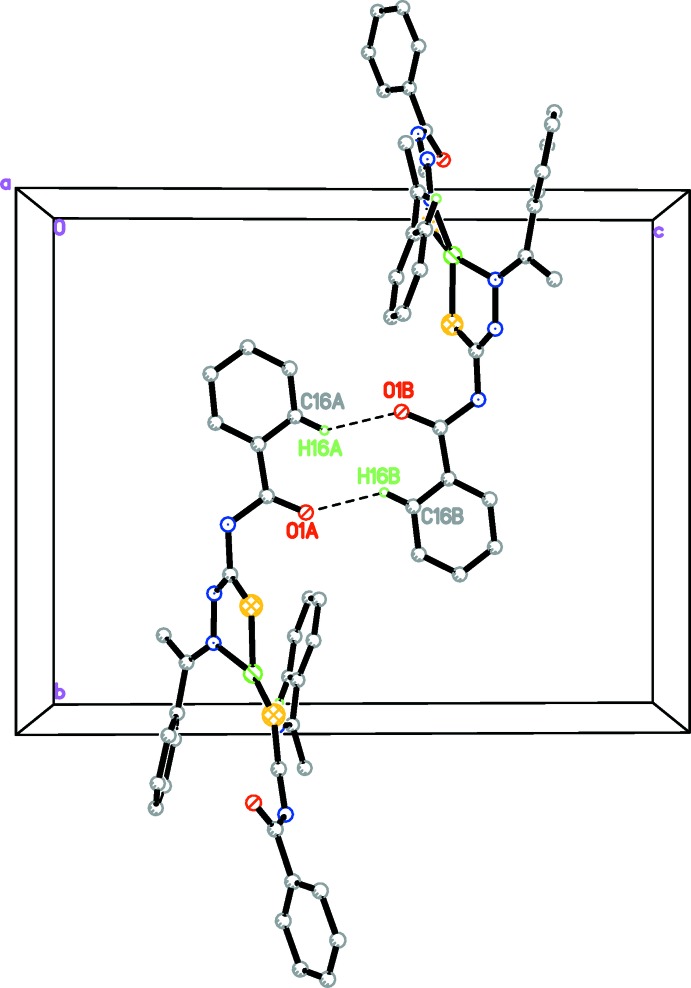
Inversion dimers found in complex (I), formed by C—H⋯O hydrogen bonds (dashed lines; see Table 2 ▸).

**Figure 4:**
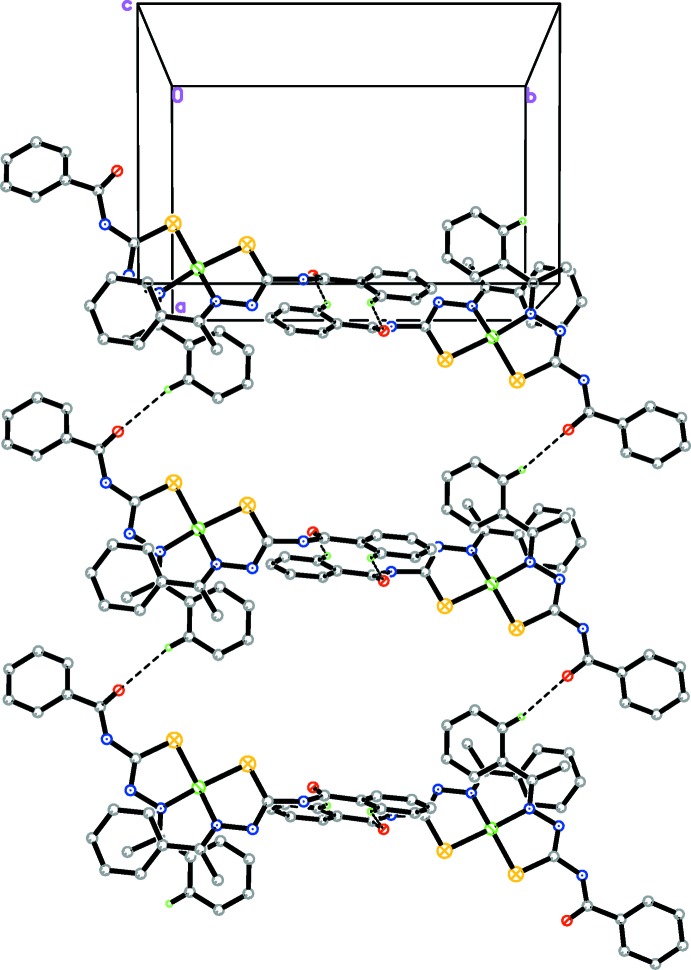
A view along the *c* axis of the crystal packing of complex (I), showing the infinite chain [100] formed by C—H⋯O inter­action (dashed lines; see Table 2 ▸). H atoms not involved in the hydrogen bonding have been omitted for clarity.

The crystal structure of (I) contains a network of C—H⋯O inter­actions (Table 2 ▸). First the inter­action C16—H16*A*⋯O1 links pairs of mol­ecules to form inversion dimers enclosing centrosymmetric 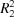
(10) ring motifs, as shown in Fig. 3 ▸. These dimers are further linked by C21—H21*A*⋯O2 inter­actions, resulting an infinite chains along [100] (Fig. 4 ▸). In addition, a C—H⋯π inter­action is also present (Table 2 ▸).

## Synthesis and crystallization   

The title complex was prepared by adding a solution of aceto­phenone-4-benzoyl-3-thio­semicarbazone (75 mg; 0.25 mmol) in di­chloro­methane (10 mL) dropwise to a stirred solution of nickel(II) nitrate hexa­hydrate (47.5 mg; 0.26 mmol) in 2-propanol (10 mL) in a small beaker. The resulting mixture solution was stirred continuously for 1 h at 318–323 K. The resultant green precipitate was separated by vacuum filtration, washed with 2-propanol and then with ether, and dried in a vacuum desiccator over dry silica gel. Single crystals suitable for X-ray analysis were obtained after slow evaporation of a di­chloro­methane solution saturated with 2-propanol. Yield; 52.5 mg, 65%. Melting point: 521–523 K.

## Refinement   

Crystal data, data collection and structure refinement details are summarized in Table 3 ▸. The H atoms attached to nitro­gen were located in difference Fourier maps and freely refined. The remaining H atoms were positioned geometrically and refined using a riding model with C—H = 0.95–0.98 Å and *U*_iso_(H) = 1.2*U*_eq_(C) or 1.5*U*_eq_(C-meth­yl). A rotating group model was applied to the methyl groups.

## Supplementary Material

Crystal structure: contains datablock(s) I. DOI: 10.1107/S2056989016006873/pj2029sup1.cif

Structure factors: contains datablock(s) I. DOI: 10.1107/S2056989016006873/pj2029Isup2.hkl

CCDC reference: 1476076

Additional supporting information:  crystallographic information; 3D view; checkCIF report

## Figures and Tables

**Table 1:** Selected geometric parameters (Å, °)

Ni1—N4	1.922 (2)	S1—C9	1.728 (3)
Ni1—N1	1.928 (2)	S2—C25	1.735 (3)
Ni1—S2	2.1489 (10)	N1—C7	1.293 (3)
Ni1—S1	2.1518 (10)	N4—C23	1.294 (3)
			
N4—Ni1—N1	101.23 (10)	N4—Ni1—S1	159.67 (7)
N4—Ni1—S2	86.18 (7)	N1—Ni1—S1	85.99 (7)
N1—Ni1—S2	159.86 (7)	S2—Ni1—S1	93.44 (4)

**Table 2:** Hydrogen-bond geometry (Å, °) *Cg*1 is the centroid of the C27–C32 ring.

*D*—H⋯*A*	*D*—H	H⋯*A*	*D*⋯*A*	*D*—H⋯*A*
C16—H16*A*⋯O1^i^	0.95	2.51	3.306 (5)	141
C21—H21*A*⋯O2^ii^	0.95	2.60	3.522 (4)	165
C19—H19*A*⋯*Cg*1^iii^	0.95	2.86	3.400 (4)	117

Symmetry codes: (i) 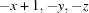; (ii) 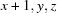; (iii) 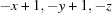.

**Table 3:** Experimental details

Crystal data	
Chemical formula	[Ni(C_16_H_14_N_3_OS)_2_]
*M*_r_	651.43
Crystal system, space group	Monoclinic, *P*2_1_/*n*
Temperature (K)	297
*a*, *b*, *c* (Å)	10.220 (3), 15.468 (5), 19.151 (6)
β (°)	92.150 (5)
*V* (Å^3^)	3025.1 (17)
*Z*	4
Radiation type	Mo *K*α
μ (mm^−1^)	0.82
Crystal size (mm)	0.19 × 0.18 × 0.09

Data collection	
Diffractometer	Bruker *APEX* DUO CCD area-detector
Absorption correction	Multi-scan (*SADABS*; Bruker, 2009 ▸)
No. of measured, independent and observed [*I* > 2σ(*I*)] reflections	43914, 5893, 4635
*R*_int_	0.070
(sin θ/λ)_max_ (Å^−1^)	0.617

Refinement	
*R*[*F* ^2^ > 2σ(*F* ^2^)], *wR*(*F* ^2^), *S*	0.048, 0.100, 1.05
No. of reflections	5893
No. of parameters	398
H-atom treatment	H atoms treated by a mixture of independent and constrained refinement
Δρ_max_, Δρ_min_ (e Å^−3^)	0.46, −0.38

Computer programs: *APEX2* and *SAINT* (Bruker, 2009 ▸), *SHELXS97* (Sheldrick, 2008 ▸), *SHELXL2014* (Sheldrick, 2015 ▸), *PLATON* (Spek, 2009 ▸) and *publCIF* (Westrip, 2010 ▸).

## supplementary crystallographic information

### Crystal data

**Table:**

[Ni(C_16_H_14_N_3_OS)_2_]	*F*(000) = 1352
*M**_r_* = 651.43	*D*_x_ = 1.430 Mg m^−^^3^
Monoclinic, *P*2_1_/*n*	Mo *K*α radiation, λ = 0.71073 Å
*a* = 10.220 (3) Å	Cell parameters from 9846 reflections
*b* = 15.468 (5) Å	θ = 2.2–30.1°
*c* = 19.151 (6) Å	µ = 0.82 mm^−^^1^
β = 92.150 (5)°	*T* = 297 K
*V* = 3025.1 (17) Å^3^	Block, dark green
*Z* = 4	0.19 × 0.18 × 0.09 mm

### Data collection

**Table:**

Bruker APEX DUO CCD area-detector diffractometer	4635 reflections with *I* > 2σ(*I*)
Radiation source: fine-focus sealed tube	*R*_int_ = 0.070
φ and ω scans	θ_max_ = 26.0°, θ_min_ = 1.7°
Absorption correction: multi-scan (*SADABS*; Bruker, 2009)	*h* = −12→12
	*k* = −19→19
43914 measured reflections	*l* = −23→23
5893 independent reflections	

### Refinement

**Table:**

Refinement on *F*^2^	0 restraints
Least-squares matrix: full	Hydrogen site location: mixed
*R*[*F*^2^ > 2σ(*F*^2^)] = 0.048	H atoms treated by a mixture of independent and constrained refinement
*wR*(*F*^2^) = 0.100	*w* = 1/[σ^2^(*F*_o_^2^) + (0.0315*P*)^2^ + 3.0091*P*] where *P* = (*F*_o_^2^ + 2*F*_c_^2^)/3
*S* = 1.05	(Δ/σ)_max_ < 0.001
5893 reflections	Δρ_max_ = 0.46 e Å^−^^3^
398 parameters	Δρ_min_ = −0.38 e Å^−^^3^

### Special details

**Table:**

Geometry. All esds (except the esd in the dihedral angle between two l.s. planes) are estimated using the full covariance matrix. The cell esds are taken into account individually in the estimation of esds in distances, angles and torsion angles; correlations between esds in cell parameters are only used when they are defined by crystal symmetry. An approximate (isotropic) treatment of cell esds is used for estimating esds involving l.s. planes.

### Fractional atomic coordinates and isotropic or equivalent isotropic displacement parameters (Å^2^)

**Table:**

	*x*	*y*	*z*	*U*_iso_*/*U*_eq_	
Ni1	0.38656 (3)	0.38363 (2)	0.14831 (2)	0.03092 (11)	
S1	0.29568 (8)	0.25816 (5)	0.14900 (5)	0.0468 (2)	
S2	0.20856 (7)	0.44818 (5)	0.11538 (4)	0.03977 (19)	
O1	0.4015 (4)	0.09249 (17)	0.07102 (13)	0.0989 (12)	
O2	0.0116 (2)	0.58787 (16)	0.14021 (16)	0.0740 (8)	
N1	0.5206 (2)	0.33342 (14)	0.20917 (11)	0.0313 (5)	
N2	0.5355 (2)	0.24301 (14)	0.20848 (12)	0.0359 (5)	
N3	0.4341 (3)	0.11462 (16)	0.18572 (14)	0.0417 (6)	
N4	0.4758 (2)	0.48357 (14)	0.11409 (11)	0.0323 (5)	
N5	0.4058 (2)	0.56192 (15)	0.10985 (12)	0.0363 (6)	
N6	0.2077 (3)	0.62374 (18)	0.09826 (14)	0.0437 (7)	
C1	0.4607 (3)	0.4993 (2)	0.27376 (15)	0.0421 (7)	
H1A	0.3864	0.4626	0.2691	0.051*	
C2	0.4439 (4)	0.5856 (2)	0.28904 (18)	0.0600 (10)	
H2A	0.3585	0.6081	0.2949	0.072*	
C3	0.5504 (5)	0.6388 (2)	0.2958 (2)	0.0709 (12)	
H3A	0.5388	0.6983	0.3061	0.085*	
C4	0.6732 (5)	0.6070 (2)	0.2879 (2)	0.0708 (12)	
H4A	0.7468	0.6444	0.2924	0.085*	
C5	0.6910 (3)	0.5205 (2)	0.27322 (18)	0.0532 (9)	
H5A	0.7770	0.4984	0.2687	0.064*	
C6	0.5840 (3)	0.46575 (18)	0.26511 (14)	0.0351 (6)	
C7	0.6017 (3)	0.37289 (18)	0.25164 (14)	0.0327 (6)	
C8	0.7109 (3)	0.3267 (2)	0.28965 (17)	0.0496 (8)	
H8A	0.7528	0.3657	0.3241	0.074*	
H8B	0.7755	0.3079	0.2563	0.074*	
H8C	0.6762	0.2761	0.3136	0.074*	
C9	0.4336 (3)	0.20575 (18)	0.18243 (14)	0.0362 (7)	
C10	0.4225 (4)	0.0628 (2)	0.12850 (16)	0.0483 (8)	
C11	0.4392 (3)	−0.03163 (18)	0.14117 (15)	0.0383 (7)	
C12	0.4104 (3)	−0.07032 (19)	0.20340 (16)	0.0422 (7)	
H12A	0.3823	−0.0362	0.2412	0.051*	
C13	0.4223 (3)	−0.1588 (2)	0.21092 (19)	0.0529 (9)	
H13A	0.4006	−0.1854	0.2537	0.063*	
C14	0.4651 (3)	−0.2085 (2)	0.1574 (2)	0.0537 (9)	
H14A	0.4727	−0.2694	0.1629	0.064*	
C15	0.4970 (4)	−0.1705 (2)	0.09600 (18)	0.0558 (9)	
H15A	0.5291	−0.2047	0.0591	0.067*	
C16	0.4826 (4)	−0.0828 (2)	0.08766 (17)	0.0568 (9)	
H16A	0.5028	−0.0569	0.0444	0.068*	
C17	0.6155 (3)	0.3332 (2)	0.06147 (14)	0.0391 (7)	
H17A	0.5243	0.3319	0.0498	0.047*	
C18	0.6894 (3)	0.2592 (2)	0.05372 (15)	0.0445 (8)	
H18A	0.6488	0.2073	0.0375	0.053*	
C19	0.8210 (3)	0.2609 (2)	0.06944 (16)	0.0495 (8)	
H19A	0.8720	0.2101	0.0643	0.059*	
C20	0.8790 (3)	0.3359 (2)	0.09259 (17)	0.0522 (9)	
H20A	0.9706	0.3369	0.1032	0.063*	
C21	0.8063 (3)	0.4102 (2)	0.10076 (16)	0.0451 (8)	
H21A	0.8481	0.4619	0.1164	0.054*	
C22	0.6723 (3)	0.40955 (19)	0.08613 (14)	0.0344 (6)	
C23	0.5946 (3)	0.48889 (18)	0.09338 (14)	0.0344 (6)	
C24	0.6553 (3)	0.5734 (2)	0.07503 (18)	0.0488 (8)	
H24A	0.6976	0.5680	0.0301	0.073*	
H24B	0.7208	0.5895	0.1114	0.073*	
H24C	0.5874	0.6180	0.0715	0.073*	
C25	0.2822 (3)	0.54878 (18)	0.10913 (14)	0.0337 (6)	
C26	0.0801 (3)	0.6392 (2)	0.11104 (17)	0.0443 (8)	
C27	0.0329 (3)	0.7260 (2)	0.08622 (15)	0.0391 (7)	
C28	0.1022 (3)	0.8009 (2)	0.10018 (17)	0.0482 (8)	
H28A	0.1824	0.7984	0.1269	0.058*	
C29	0.0558 (4)	0.8797 (2)	0.07568 (18)	0.0590 (9)	
H29A	0.1040	0.9310	0.0859	0.071*	
C30	−0.0587 (4)	0.8839 (3)	0.0369 (2)	0.0629 (10)	
H30A	−0.0902	0.9381	0.0199	0.075*	
C31	−0.1284 (4)	0.8099 (3)	0.02259 (18)	0.0610 (10)	
H31A	−0.2082	0.8130	−0.0044	0.073*	
C32	−0.0837 (3)	0.7306 (2)	0.04694 (17)	0.0501 (8)	
H32A	−0.1326	0.6796	0.0368	0.060*	
H1N3	0.469 (3)	0.094 (2)	0.2191 (16)	0.040 (9)*	
H1N6	0.247 (3)	0.662 (2)	0.0807 (18)	0.057 (11)*	

### Atomic displacement parameters (Å^2^)

**Table:**

	*U*^11^	*U*^22^	*U*^33^	*U*^12^	*U*^13^	*U*^23^
Ni1	0.0308 (2)	0.02381 (19)	0.0382 (2)	−0.00201 (15)	0.00162 (14)	0.00350 (15)
S1	0.0369 (4)	0.0279 (4)	0.0755 (6)	−0.0047 (3)	−0.0012 (4)	0.0022 (4)
S2	0.0331 (4)	0.0334 (4)	0.0527 (4)	−0.0017 (3)	0.0002 (3)	0.0065 (3)
O1	0.209 (4)	0.0493 (16)	0.0385 (13)	0.043 (2)	0.0135 (18)	0.0059 (12)
O2	0.0527 (16)	0.0495 (15)	0.122 (2)	0.0062 (12)	0.0323 (16)	0.0261 (15)
N1	0.0352 (13)	0.0235 (12)	0.0354 (12)	0.0022 (10)	0.0030 (10)	0.0011 (9)
N2	0.0444 (15)	0.0225 (13)	0.0408 (13)	0.0024 (11)	0.0020 (11)	0.0013 (10)
N3	0.0586 (18)	0.0232 (13)	0.0430 (15)	0.0007 (12)	−0.0011 (13)	0.0044 (12)
N4	0.0314 (13)	0.0277 (13)	0.0378 (12)	0.0001 (10)	0.0020 (10)	0.0028 (10)
N5	0.0344 (14)	0.0286 (13)	0.0462 (14)	0.0024 (11)	0.0049 (11)	0.0073 (10)
N6	0.0382 (16)	0.0345 (15)	0.0592 (17)	0.0043 (13)	0.0111 (13)	0.0155 (13)
C1	0.0466 (19)	0.0400 (18)	0.0397 (16)	0.0040 (15)	0.0008 (14)	−0.0050 (13)
C2	0.076 (3)	0.048 (2)	0.056 (2)	0.021 (2)	−0.0094 (19)	−0.0111 (17)
C3	0.108 (4)	0.032 (2)	0.070 (2)	0.009 (2)	−0.031 (2)	−0.0107 (17)
C4	0.086 (3)	0.040 (2)	0.083 (3)	−0.020 (2)	−0.026 (2)	0.0003 (19)
C5	0.050 (2)	0.045 (2)	0.064 (2)	−0.0099 (16)	−0.0082 (16)	−0.0020 (16)
C6	0.0408 (17)	0.0321 (16)	0.0321 (14)	−0.0011 (13)	−0.0021 (12)	−0.0003 (12)
C7	0.0335 (15)	0.0321 (16)	0.0326 (14)	0.0006 (13)	0.0046 (12)	0.0008 (12)
C8	0.0474 (19)	0.045 (2)	0.0550 (19)	0.0114 (16)	−0.0114 (15)	−0.0040 (15)
C9	0.0460 (18)	0.0242 (15)	0.0389 (15)	0.0008 (13)	0.0093 (13)	0.0014 (12)
C10	0.072 (2)	0.0336 (18)	0.0398 (17)	0.0091 (16)	0.0133 (16)	0.0018 (14)
C11	0.0477 (18)	0.0260 (15)	0.0411 (16)	0.0049 (13)	0.0005 (13)	−0.0012 (12)
C12	0.0474 (19)	0.0321 (17)	0.0477 (17)	0.0006 (14)	0.0103 (14)	0.0012 (13)
C13	0.052 (2)	0.0371 (19)	0.070 (2)	−0.0007 (16)	0.0124 (17)	0.0146 (17)
C14	0.051 (2)	0.0260 (17)	0.083 (3)	−0.0021 (15)	−0.0117 (18)	−0.0024 (17)
C15	0.072 (2)	0.040 (2)	0.055 (2)	0.0129 (17)	−0.0132 (18)	−0.0175 (16)
C16	0.089 (3)	0.043 (2)	0.0389 (17)	0.0126 (19)	−0.0001 (17)	−0.0044 (15)
C17	0.0364 (16)	0.0485 (19)	0.0327 (14)	−0.0001 (14)	0.0056 (12)	−0.0008 (13)
C18	0.054 (2)	0.0422 (19)	0.0374 (16)	0.0025 (16)	0.0067 (14)	−0.0032 (13)
C19	0.054 (2)	0.051 (2)	0.0435 (17)	0.0151 (17)	0.0088 (15)	0.0042 (15)
C20	0.0327 (17)	0.067 (2)	0.057 (2)	0.0062 (17)	0.0055 (15)	0.0032 (18)
C21	0.0358 (17)	0.049 (2)	0.0511 (18)	−0.0033 (15)	0.0059 (14)	0.0011 (15)
C22	0.0340 (16)	0.0389 (17)	0.0309 (14)	−0.0002 (13)	0.0074 (12)	0.0039 (12)
C23	0.0341 (16)	0.0353 (16)	0.0340 (14)	−0.0030 (13)	0.0026 (12)	0.0041 (12)
C24	0.0421 (19)	0.0419 (19)	0.063 (2)	−0.0059 (15)	0.0133 (16)	0.0101 (16)
C25	0.0370 (17)	0.0304 (16)	0.0341 (14)	0.0039 (13)	0.0068 (12)	0.0065 (12)
C26	0.0406 (18)	0.0385 (18)	0.0542 (19)	0.0018 (14)	0.0093 (15)	0.0042 (14)
C27	0.0345 (16)	0.0404 (18)	0.0431 (16)	0.0049 (14)	0.0101 (13)	0.0025 (13)
C28	0.049 (2)	0.044 (2)	0.0521 (18)	0.0039 (16)	−0.0020 (15)	−0.0012 (15)
C29	0.077 (3)	0.038 (2)	0.062 (2)	0.0052 (19)	0.006 (2)	−0.0033 (16)
C30	0.075 (3)	0.050 (2)	0.064 (2)	0.022 (2)	0.009 (2)	0.0128 (18)
C31	0.047 (2)	0.079 (3)	0.057 (2)	0.018 (2)	0.0037 (17)	0.0150 (19)
C32	0.0397 (18)	0.053 (2)	0.058 (2)	−0.0003 (16)	0.0060 (15)	0.0043 (16)

### Geometric parameters (Å, º)

**Table:**

Ni1—N4	1.922 (2)	C11—C16	1.381 (4)
Ni1—N1	1.928 (2)	C12—C13	1.381 (4)
Ni1—S2	2.1489 (10)	C12—H12A	0.9500
Ni1—S1	2.1518 (10)	C13—C14	1.366 (5)
S1—C9	1.728 (3)	C13—H13A	0.9500
S2—C25	1.735 (3)	C14—C15	1.365 (5)
O1—C10	1.204 (4)	C14—H14A	0.9500
O2—C26	1.210 (4)	C15—C16	1.374 (5)
N1—C7	1.293 (3)	C15—H15A	0.9500
N1—N2	1.407 (3)	C16—H16A	0.9500
N2—C9	1.275 (4)	C17—C18	1.383 (4)
N3—C10	1.359 (4)	C17—C22	1.391 (4)
N3—C9	1.411 (4)	C17—H17A	0.9500
N3—H1N3	0.78 (3)	C18—C19	1.367 (4)
N4—C23	1.294 (3)	C18—H18A	0.9500
N4—N5	1.408 (3)	C19—C20	1.369 (5)
N5—C25	1.279 (4)	C19—H19A	0.9500
N6—C26	1.356 (4)	C20—C21	1.381 (5)
N6—C25	1.399 (4)	C20—H20A	0.9500
N6—H1N6	0.80 (3)	C21—C22	1.387 (4)
C1—C2	1.379 (5)	C21—H21A	0.9500
C1—C6	1.379 (4)	C22—C23	1.471 (4)
C1—H1A	0.9500	C23—C24	1.494 (4)
C2—C3	1.366 (6)	C24—H24A	0.9800
C2—H2A	0.9500	C24—H24B	0.9800
C3—C4	1.361 (6)	C24—H24C	0.9800
C3—H3A	0.9500	C26—C27	1.497 (4)
C4—C5	1.380 (5)	C27—C28	1.380 (4)
C4—H4A	0.9500	C27—C32	1.387 (4)
C5—C6	1.388 (4)	C28—C29	1.383 (5)
C5—H5A	0.9500	C28—H28A	0.9500
C6—C7	1.472 (4)	C29—C30	1.364 (5)
C7—C8	1.491 (4)	C29—H29A	0.9500
C8—H8A	0.9800	C30—C31	1.371 (5)
C8—H8B	0.9800	C30—H30A	0.9500
C8—H8C	0.9800	C31—C32	1.383 (5)
C10—C11	1.490 (4)	C31—H31A	0.9500
C11—C12	1.375 (4)	C32—H32A	0.9500
			
N4—Ni1—N1	101.23 (10)	C14—C13—H13A	119.7
N4—Ni1—S2	86.18 (7)	C12—C13—H13A	119.7
N1—Ni1—S2	159.86 (7)	C15—C14—C13	119.9 (3)
N4—Ni1—S1	159.67 (7)	C15—C14—H14A	120.1
N1—Ni1—S1	85.99 (7)	C13—C14—H14A	120.1
S2—Ni1—S1	93.44 (4)	C14—C15—C16	119.8 (3)
C9—S1—Ni1	94.53 (10)	C14—C15—H15A	120.1
C25—S2—Ni1	94.14 (10)	C16—C15—H15A	120.1
C7—N1—N2	114.1 (2)	C15—C16—C11	121.1 (3)
C7—N1—Ni1	127.91 (19)	C15—C16—H16A	119.5
N2—N1—Ni1	118.01 (17)	C11—C16—H16A	119.5
C9—N2—N1	111.4 (2)	C18—C17—C22	121.1 (3)
C10—N3—C9	123.6 (3)	C18—C17—H17A	119.4
C10—N3—H1N3	116 (2)	C22—C17—H17A	119.4
C9—N3—H1N3	116 (2)	C19—C18—C17	119.8 (3)
C23—N4—N5	114.1 (2)	C19—C18—H18A	120.1
C23—N4—Ni1	128.18 (19)	C17—C18—H18A	120.1
N5—N4—Ni1	117.74 (17)	C18—C19—C20	119.9 (3)
C25—N5—N4	111.3 (2)	C18—C19—H19A	120.1
C26—N6—C25	129.9 (3)	C20—C19—H19A	120.1
C26—N6—H1N6	117 (3)	C19—C20—C21	120.9 (3)
C25—N6—H1N6	113 (3)	C19—C20—H20A	119.5
C2—C1—C6	120.8 (3)	C21—C20—H20A	119.5
C2—C1—H1A	119.6	C20—C21—C22	120.2 (3)
C6—C1—H1A	119.6	C20—C21—H21A	119.9
C3—C2—C1	119.8 (4)	C22—C21—H21A	119.9
C3—C2—H2A	120.1	C21—C22—C17	118.1 (3)
C1—C2—H2A	120.1	C21—C22—C23	120.5 (3)
C4—C3—C2	120.5 (3)	C17—C22—C23	121.4 (3)
C4—C3—H3A	119.8	N4—C23—C22	119.5 (2)
C2—C3—H3A	119.8	N4—C23—C24	122.0 (3)
C3—C4—C5	120.1 (4)	C22—C23—C24	118.5 (2)
C3—C4—H4A	119.9	C23—C24—H24A	109.5
C5—C4—H4A	119.9	C23—C24—H24B	109.5
C4—C5—C6	120.3 (4)	H24A—C24—H24B	109.5
C4—C5—H5A	119.8	C23—C24—H24C	109.5
C6—C5—H5A	119.8	H24A—C24—H24C	109.5
C1—C6—C5	118.5 (3)	H24B—C24—H24C	109.5
C1—C6—C7	120.5 (3)	N5—C25—N6	113.7 (3)
C5—C6—C7	120.9 (3)	N5—C25—S2	124.9 (2)
N1—C7—C6	119.4 (2)	N6—C25—S2	121.2 (2)
N1—C7—C8	122.1 (3)	O2—C26—N6	123.0 (3)
C6—C7—C8	118.5 (2)	O2—C26—C27	123.4 (3)
C7—C8—H8A	109.5	N6—C26—C27	113.7 (3)
C7—C8—H8B	109.5	C28—C27—C32	119.1 (3)
H8A—C8—H8B	109.5	C28—C27—C26	122.3 (3)
C7—C8—H8C	109.5	C32—C27—C26	118.6 (3)
H8A—C8—H8C	109.5	C27—C28—C29	120.5 (3)
H8B—C8—H8C	109.5	C27—C28—H28A	119.7
N2—C9—N3	115.7 (3)	C29—C28—H28A	119.7
N2—C9—S1	125.1 (2)	C30—C29—C28	120.2 (4)
N3—C9—S1	119.1 (2)	C30—C29—H29A	119.9
O1—C10—N3	121.3 (3)	C28—C29—H29A	119.9
O1—C10—C11	122.5 (3)	C29—C30—C31	119.8 (3)
N3—C10—C11	116.2 (3)	C29—C30—H30A	120.1
C12—C11—C16	118.6 (3)	C31—C30—H30A	120.1
C12—C11—C10	122.7 (3)	C30—C31—C32	120.7 (3)
C16—C11—C10	118.6 (3)	C30—C31—H31A	119.7
C11—C12—C13	120.0 (3)	C32—C31—H31A	119.7
C11—C12—H12A	120.0	C31—C32—C27	119.7 (3)
C13—C12—H12A	120.0	C31—C32—H32A	120.2
C14—C13—C12	120.6 (3)	C27—C32—H32A	120.2
			
C7—N1—N2—C9	161.4 (2)	C12—C11—C16—C15	0.0 (5)
Ni1—N1—N2—C9	−19.0 (3)	C10—C11—C16—C15	−178.6 (3)
C23—N4—N5—C25	159.4 (2)	C22—C17—C18—C19	−1.0 (4)
Ni1—N4—N5—C25	−20.2 (3)	C17—C18—C19—C20	−0.2 (5)
C6—C1—C2—C3	0.0 (5)	C18—C19—C20—C21	0.4 (5)
C1—C2—C3—C4	−0.3 (6)	C19—C20—C21—C22	0.6 (5)
C2—C3—C4—C5	−0.3 (6)	C20—C21—C22—C17	−1.7 (4)
C3—C4—C5—C6	1.2 (6)	C20—C21—C22—C23	−178.8 (3)
C2—C1—C6—C5	1.0 (4)	C18—C17—C22—C21	1.9 (4)
C2—C1—C6—C7	177.5 (3)	C18—C17—C22—C23	179.0 (2)
C4—C5—C6—C1	−1.6 (5)	N5—N4—C23—C22	−171.8 (2)
C4—C5—C6—C7	−178.1 (3)	Ni1—N4—C23—C22	7.8 (4)
N2—N1—C7—C6	−171.0 (2)	N5—N4—C23—C24	7.0 (4)
Ni1—N1—C7—C6	9.4 (4)	Ni1—N4—C23—C24	−173.5 (2)
N2—N1—C7—C8	6.9 (4)	C21—C22—C23—N4	−145.6 (3)
Ni1—N1—C7—C8	−172.7 (2)	C17—C22—C23—N4	37.4 (4)
C1—C6—C7—N1	41.4 (4)	C21—C22—C23—C24	35.6 (4)
C5—C6—C7—N1	−142.2 (3)	C17—C22—C23—C24	−141.4 (3)
C1—C6—C7—C8	−136.6 (3)	N4—N5—C25—N6	−174.2 (2)
C5—C6—C7—C8	39.8 (4)	N4—N5—C25—S2	1.9 (3)
N1—N2—C9—N3	−174.0 (2)	C26—N6—C25—N5	−161.8 (3)
N1—N2—C9—S1	2.2 (3)	C26—N6—C25—S2	21.9 (5)
C10—N3—C9—N2	−121.1 (3)	Ni1—S2—C25—N5	13.3 (3)
C10—N3—C9—S1	62.5 (4)	Ni1—S2—C25—N6	−170.8 (2)
Ni1—S1—C9—N2	12.1 (3)	C25—N6—C26—O2	5.6 (6)
Ni1—S1—C9—N3	−171.8 (2)	C25—N6—C26—C27	−174.9 (3)
C9—N3—C10—O1	−5.5 (6)	O2—C26—C27—C28	131.8 (4)
C9—N3—C10—C11	173.7 (3)	N6—C26—C27—C28	−47.7 (4)
O1—C10—C11—C12	−152.7 (4)	O2—C26—C27—C32	−49.2 (5)
N3—C10—C11—C12	28.1 (5)	N6—C26—C27—C32	131.3 (3)
O1—C10—C11—C16	25.8 (6)	C32—C27—C28—C29	0.3 (5)
N3—C10—C11—C16	−153.4 (3)	C26—C27—C28—C29	179.3 (3)
C16—C11—C12—C13	−1.4 (5)	C27—C28—C29—C30	−0.4 (5)
C10—C11—C12—C13	177.1 (3)	C28—C29—C30—C31	0.3 (6)
C11—C12—C13—C14	1.3 (5)	C29—C30—C31—C32	−0.1 (6)
C12—C13—C14—C15	0.3 (5)	C30—C31—C32—C27	−0.1 (5)
C13—C14—C15—C16	−1.7 (5)	C28—C27—C32—C31	0.0 (5)
C14—C15—C16—C11	1.6 (6)	C26—C27—C32—C31	−179.1 (3)

### Hydrogen-bond geometry (Å, º)

Cg1 is the centroid of the C27–C32 ring.

**Table:**

*D*—H···*A*	*D*—H	H···*A*	*D*···*A*	*D*—H···*A*
C16—H16*A*···O1^i^	0.95	2.51	3.306 (5)	141
C21—H21*A*···O2^ii^	0.95	2.60	3.522 (4)	165
C19—H19*A*···*Cg*1^iii^	0.95	2.86	3.400 (4)	117

Symmetry codes: (i) −*x*+1, −*y*, −*z*; (ii) *x*+1, *y*, *z*; (iii) −*x*+1, −*y*+1, −*z*.
